# Pyogranulomatous dermatitis associated with *Sporobolomyces roseus* in a dog

**DOI:** 10.1186/s12917-026-05576-8

**Published:** 2026-05-22

**Authors:** Sarah Berger, David Walker, Mutien-Marie Garigliany, Isabelle Remy, Elodie Roels

**Affiliations:** 1 Department of Clinical Sciences, Faculty of Veterinary Medicine, University of Liege, Liege, 4000 Belgium; 2 Department of Dermatology, National Veterinary School of Alfort, Maisons-Alfort, 94700 France; 3 Veterinary Pathology Group, Bristol, BS16 1EJ United Kingdom; 4 Laboratory of Veterinary Pathology, Faculty of Veterinary Medicine, INDEEP, University of Liege, Liege, 4000 Belgium

**Keywords:** Skin, Dermis, Cutaneous fungus, Canine, Yeast, Dermatology

## Abstract

**Background:**

*Sporobolomyces* spp. is a yeast-like fungal organism commonly found in the environment. It has been sporadically reported in human medicine as a potential cause of dermatitis, lymphadenitis, meningitis, and endophthalmitis. In veterinary medicine, this fungus has only been described once in a dog affected with granulomatous meningoencephalitis.

**Case presentation:**

A 3-year-old Jagd Terrier was presented with crusted, erosive and ulcerative skin lesions primarily affecting the nose, but also the limbs and tail. A pyogranulomatous dermatitis was identified on skin histopathological sections. Pan-fungal conventional polymerase chain reaction (PCR) performed on skin biopsies yielded a positive result. Subsequent sequencing of the PCR product identified *Sporobolomyces roseus* with 99.27% sequence identity. Anti-fungal treatment with a combination of voriconazole and terbinafine for two months led to an almost complete resolution of the lesions. For financial reasons, antifungal therapy was discontinued prematurely, leading to a relapse of dermatological lesions. Other anti-fungal treatments were attempted without clinical improvement leading to subsequent euthanasia of the dog.

**Conclusions:**

This case report described dermatological lesions associated with *Sporobolomyces roseus* in a dog and the challenges associated with anti-fungal treatment.

## Background

The *Sporobolomyces* are yeast-like fungal organisms belonging to the *Sporobolomycetaceae* family [1]. Phylogenetically, they are closely related to *Rhodotorula* spp [2, 3]. There are 53 known species of *Sporobolomyces*, which are commonly found in nature, particularly on leaf surfaces, as well as in soil, fruits, and indoor air, especially during the summer season [2, 3].

Among these 53 species, three have been reported as pathological in humans : *Sporobolomyces roseus*, *Sporobolomyces salmonicolor* and *Sporobolomyces holsaticus* [1]. Half of the reported cases of *Sporobolomyces* spp. infections in humans occurred in immunocompromised patients, either affected with human immunodeficiency virus or suffering from diabetes mellitus [2, 3]. People with a competent immune system can also be affected [3, 4]. The different types of infections reported include dermatitis primarily affecting the hands and lower extremities, lymphadenitis, meningitis and endophthalmitis [3–5]. In comparison with humans, only one case of *Sporobolomyces* spp. infection has been reported in veterinary medicine [6]. It involved a dog, with no immune dysfunction, diagnosed with granulomatous meningoencephalitis caused by *Sporobolomyces roseus* [6].

In human medicine, *Sporobolomyces* spp. is diagnosed through histopathological examination of biopsies samples [4, 5, 7]. Fungal elements are primarily detected using Periodic Acid-Schiff (PAS) and Grocott’s Methenamine Silver (GMS) stains [6, 8]. Identification is then confirmed by culture, as *Sporobolomyces* colonies usually appear red to orange, resembling those of *Rhodotorula* spp [2]. Alternatively, identification can be performed using pan-fungal polymerase chain reaction (PCR) followed by sequence analysis of the D1/D2 domains or the internal transcribed spacer regions ITS1 and ITS2 of the fungal rDNA [2].

The present case report described a case of dermatological lesions associated with *Sporobolomyces roseus* in a dog and outlined the therapeutic approach and outcome.

## Case presentation

A three-year-old entire female hunting Jagd Terrier was presented with a five-month history of non-pruritic skin lesions, predominantly affecting the nose, with additional lesions present on both stifles. The dog also presented intermittent sneezing and inspiratory stertor associated with an abundant bilateral serous nasal discharge. No other symptoms were reported. The dog lived exclusively outdoors in Belgium, in a rural environment, and was frequently in contact with other dogs and wildlife during hunting sessions. The dog had no travel history and was up to date with vaccinations and deworming.

Various empirical treatments had been attempted at a local veterinary practice before referral, including doxycycline, prednisolone, ketoconazole, and topical ointments, without improvement. The exact administered dosages for most medications were not available except for prednisolone which was prescribed at 1 mg/kg once a day for a week, then tapered down over three weeks, then stopped. At no point did the owners administer any treatment on their own initiative. Initial blood work performed before any treatment showed no abnormalities. A fungal culture performed on hair samples taken from the stifle was negative.

**Fig. 1:**
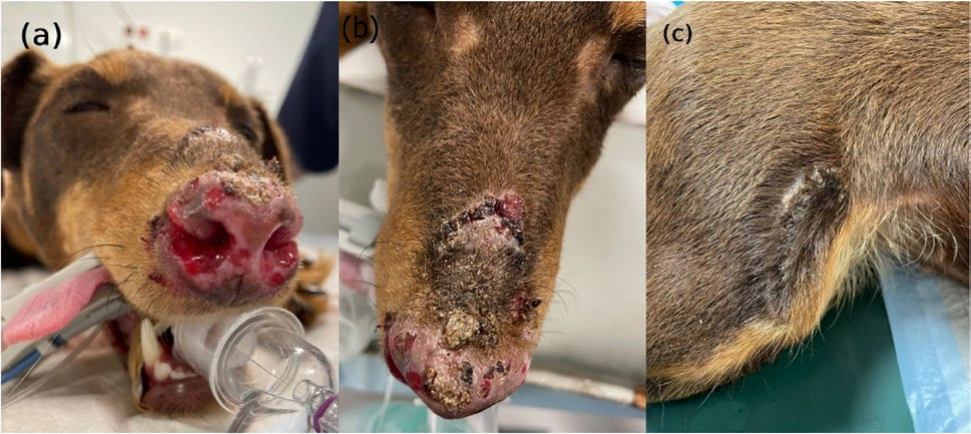
Dermatological lesions (**a**) swelling and depigmentation of the nasal planum and bilateral deep ulcerations of the ventral edge of the nostrils; (**b**) crusted area on the nasal planum and the haired bridge of the muzzle; (**c**) oval, partially alopecic crusted lesions on the right stifle

On physical examination, deep ulcerations were identified bilaterally in the ventral edge of the nostrils, along with depigmentation and severe swelling of the nasal planum responsible for a significant narrowing of the nostrils (Fig. 1a). The nasal planum lost the typical “cobblestone” appearance. Crusted lesions were present on the haired bridge of the muzzle and dorsal nasal planum (Fig. 1b). Oval, partially alopecic crusted lesions were also observed on both stifles (Fig. 1c). A focal alopecic and crusted lesion was also noted on the right tarsus and on the tail. The rest of the physical examination was within normal limits; notably there was no enlargement of peripheral lymph nodes.

An anterograde rhinoscopy performed under general anesthesia revealed bilateral narrowing of the nasal vestibule due to swelling of the nasal wings and septum; rest of the rhinoscopic examination was within normal limits. Retrograde rhinoscopy indicated mild follicular hyperplasia of the nasopharyngeal tonsil.

**Fig. 2:**
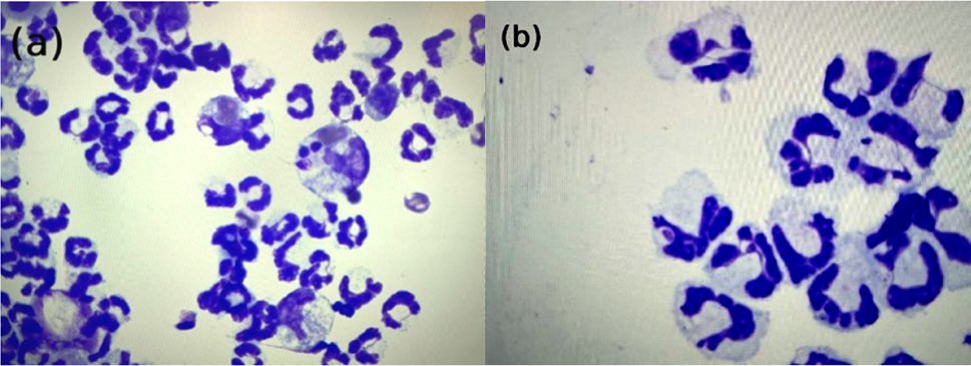
**a-b** Cytological evaluation of a nasal planum impression smear reveals a pyogranulomatous inflammation with numerous polymorphonuclear cells and occasional macrophages. No infectious agents were detected

Cytological examination of an impression smear from the dermatological lesions on the nasal planum revealed a pyogranulomatous inflammation with no visible infectious agent (Fig. 2a and b).

**Fig. 3:**
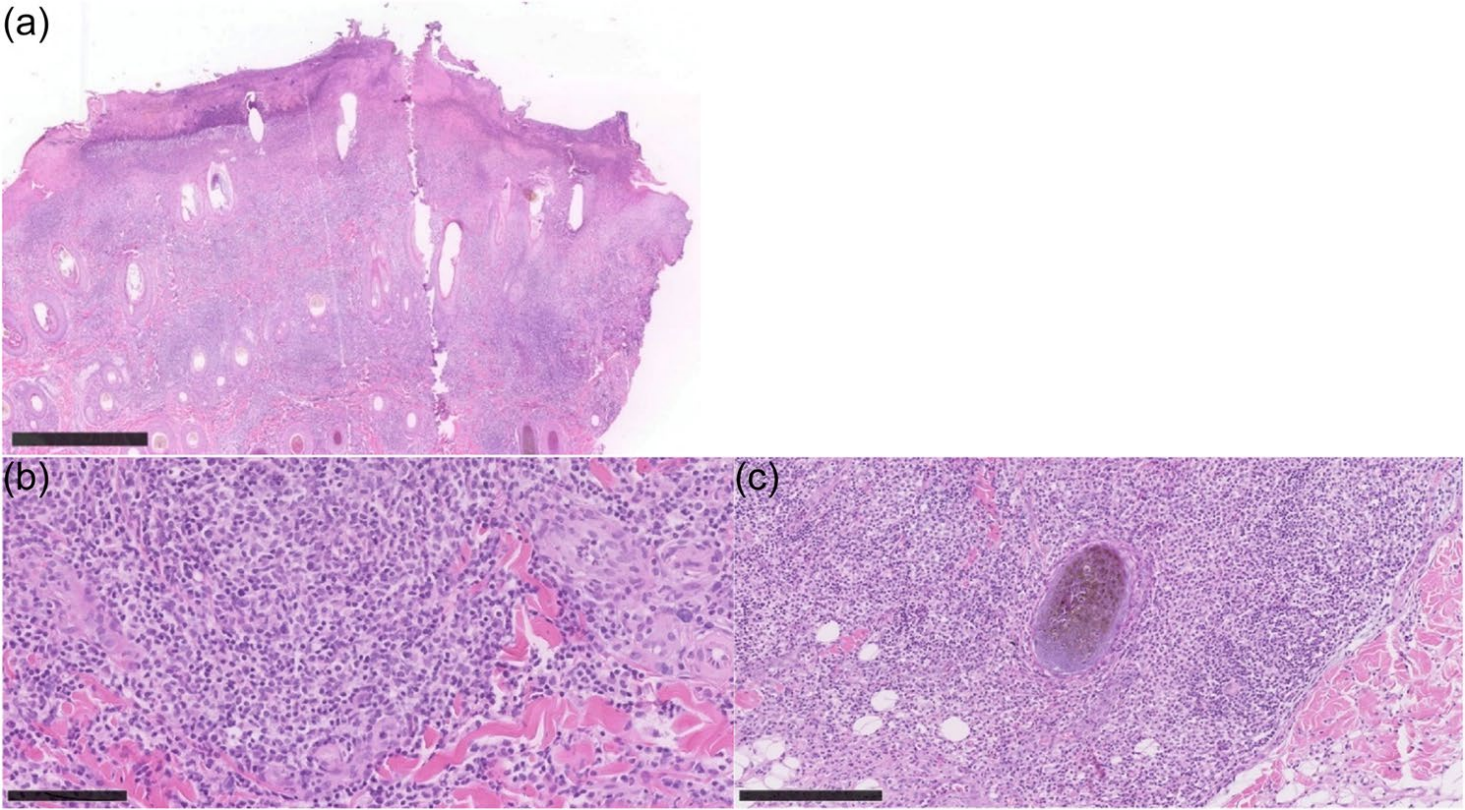
Histological analysis of biopsy lesions stained with hematoxylin and eosin (**a**, **b**, **c**). **a** low magnification image of the skin biopsy. The dermis is prominently disrupted and expanded by chronic inflammation, including within periadnexal regions; scale bar: 1 mm (**b**) a loose focus of mixed, predominantly granulomatous, inflammation effacing the deep dermis of the haired skin; scale bar: 100 μm (**c**) a hair follicle is surrounded by marked mixed inflammatory infiltrates, with a predominance of macrophages, and fewer neutrophils, lymphocytes and plasma cells; scale bar: 250 μm

Five 4-mm punch biopsies of the skin lesions were taken and submitted for histopathological examination, including two from the nasal lesions and three from the stifles and tarsus. Biopsies specimens were routinely processed and stained with hematoxylin and eosin (HE). The histopathological sections revealed an extensive, chronic, pyogranulomatous inflammatory infiltrate within the dermis and deep dermis (Fig. 3a and b). There was a marked granulomatous to pyogranulomatous inflammatory infiltrate around the hair follicles (Fig. 3c). No infectious organisms were observed within HE-stained sections. PAS and GMS staining did not detect the presence of fungal organisms.

Given the presence of a pyogranulomatous inflammation on both cytology and histopathology, a PCR panel for infectious agents was performed on a biopsy sample that was prospectively stored snap-frozen at -80°C pending reception of the histopathological results. The PCRs performed included: pan-fungal PCR, *Mycobacteria* spp., *Bartonella* spp., *Neospora canis*, and *Sporothrix* spp PCRs. The pan-fungal PCR was performed according to the method described by Henry, Iwen, and Hinrichs [9]. Two fungal oligonucleotide primers targeting the ITS region were used for amplification: ITS1 (5’-TCCGTAGGTGAACCTGCGG-3’) and ITS4 (5’-TCCTCCGCTTATTGATATG-3’). The PCR assay was carried out using the Luna Universal Probe qPCR Mix in a total reaction volume of 20 µL containing 2 µL of template DNA, 10 µL of buffer, 1 µL of forward primer, 1 µL of reverse primer, and 6 µL of water. Amplification was performed with an initial denaturation at 95 °C for 5 min, followed by 45 cycles of denaturation at 95 °C for 30 s, annealing at 50 °C for 30 s, and extension at 72 °C for 1 min. A final extension step at 72 °C for 3 min was carried out after the last cycle. The PCR products were then analyzed on a 2% agarose gel, with an expected fragment size of 550 bp. Conventional pan-fungal PCR was positive, other PCRs were negative. Sequence analysis of the PCR product was compatible with *Sporobolomyces roseus* (99,27% identity). The DNA sequences characterized are available on GenBank under accession numbers PZ273470.

**Fig. 4:**
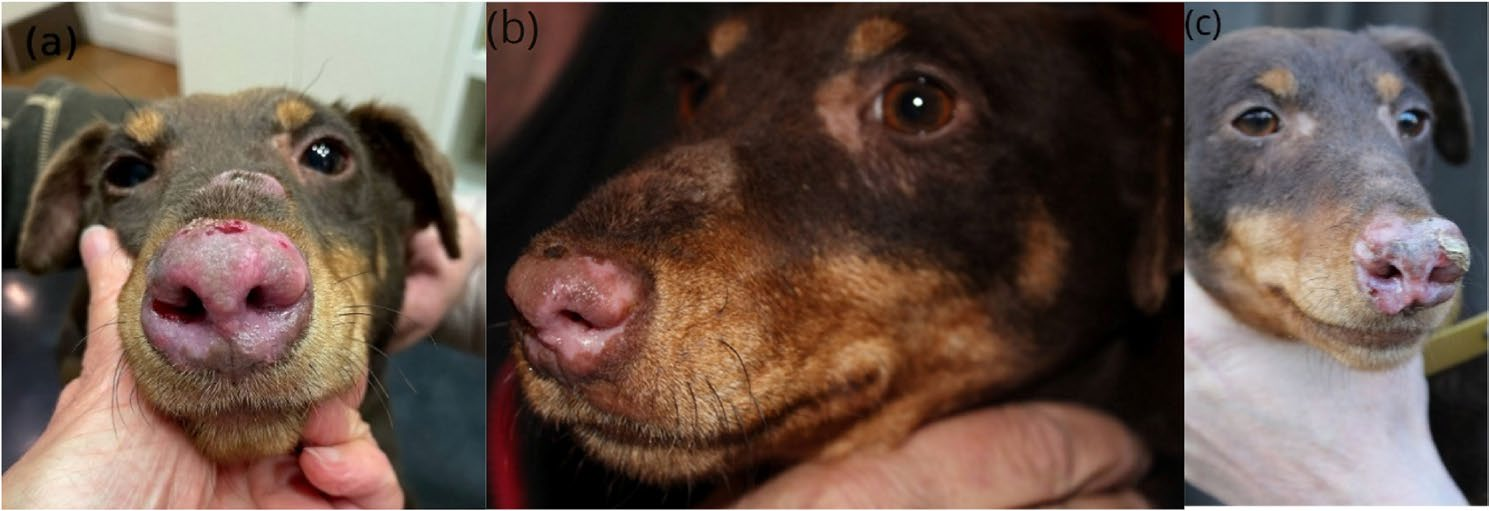
Evolution of lesions during treatment. **a** After one month of combined voriconazole and terbinafine treatment, there was an almost complete disappearance of nostril ulcers, reduced nasal swelling, and resolution of crusted areas on the muzzle; (**b**) two months into the treatment, swelling had subsided, with only mild depigmentation remaining and no crusted areas on the nasal planum; (**c**) four months after diagnosis, anti-fungal therapy having been stopped for one month, reoccurrence of swelling, crusts and loss of the normal “cobblestone” architecture of the nasal planum

The dog was treated orally with voriconazole 5.5 mg/kg (Vfend 40 mg/ml oral solution; Pfizer) twice daily and terbinafine 34 mg/kg (Terbinafine 250 mg; EG) twice daily for four weeks. After one month, a significant improvement of the lesions was observed. There was almost a complete resolution of the nostril ulcers, but the nasal swelling persisted. There was also a disappearance of the crusted area on the haired bridge of the muzzle (Fig. 4a). After two months of treatment, a good improvement of the respiratory and cutaneous symptoms was observed. There was no more sneezing or nasal discharge. The swelling of the nasal planum had completely resolved, although a mild depigmentation persisted (Fig. 4b).

Voriconazole treatment was prematurely discontinued by the owners after two months due to financial constraints. Terbinafine was then continued as a monotherapy for another month before also being stopped for the same reasons.

One month after the complete cessation of antifungal treatment, the dog experienced a relapse, developing cutaneous lesions characterized by swelling, crusting, and loss of the normal “cobblestone” architecture of the nasal planum (Fig. 4c). Itraconazole at 15 mg/kg once daily (Sporanox 100 mg ; Janssen Pharmaceutica) was administered for one month but failed to improve the lesions. The treatment was then changed to corticosteroids at 0,8 mg/kg once daily (Prednicortone 5 mg ; Dechra Veterinary Products SAS) for two weeks and fluconazole at 6 mg/kg twice daily (Diflucan 200 mg/5 ml ; Pfizer) for two months. However, adherence to the treatment was inconsistent due to financial limitations. The dermatological condition deteriorated further, leading to extensive ulceration of the nasal planum, severe edema, and erosion of the muzzle. Crusted and erosive lesions also worsened on the haired bridge of the muzzle, the periocular region, the right hind limb, and the tail.

Additionally, the dog’s general condition progressively deteriorated, with the development of lethargy and anorexia. Given the severity of the disease, the associated impairment of the dog’s quality of life, and the poor prognosis, the dog was humanely euthanized eight months after diagnosis at the referring veterinarian’s clinic. Euthanasia was carried out via the intravenous route. The protocol consisted of sedation with medetomidine at a dose of 100 µg/kg (Sedator 1 mg/mL, Dechra) combined with butorphanol at 0.2 mg/kg (Morphasol 4 mg/mL, Dechra), followed by the administration of sodium pentobarbital at a dose of 182 mg/kg (Dolethal 200 mg/mL, Vetoquinol).

### Discussion and conclusions

In the present case report, the dog was initially presented with dermatological lesions located on the extremities, including the nasal bridge, nasal planum, limbs, and tail. The dog was considered immunocompetent based on unremarkable hematological findings, the absence of identified concurrent disease, and no history of immunosuppressive medication. In human medicine, dermatological cases associated with *Sporobolomyces* spp. have been reported as localized lesions on the hands [1, 3]. Both human published cases reported extensive scaly plaques with erythema and excoriations in immunocompetent individuals, which is consistent with the present case. In humans, it was hypothesized that micro-traumas facilitated fungal penetration into deeper layers of the skin [1]. In the current case, the dog’s hunting behavior may have led to micro-traumatic skin injuries on the face, thereby facilitating the introduction of *Sporobolomyces roseus* into the dermis.

Cytological and histopathological examinations revealed a pyogranulomatous dermatitis without identifiable micro-organisms. Differential diagnoses for such lesions include deep mycoses, deep atypical dermatological infections, or sterile pyogranulomatous syndromes [10]. The latter could have responded to immunomodulatory therapy. However, corticosteroid treatment at 1 mg/kg once a day with tapering dose was attempted before referral early in the course of the disease without any improvement, supporting the hypothesis of a deep fungal or atypical infection in this case.

To date, the only reported case in veterinary medicine of *Sporobolomyces* infection involved a dog with rapidly progressive neurological signs caused by granulomatous meningoencephalitis due to *Sporobolomyces roseus*, diagnosed post-mortem [6]. Given the rarity of such cases, no standardized therapeutic protocol has been established in veterinary medicine, and the optimal treatment duration remains unknown. In human medicine, the type of treatment and the duration vary across the scientific literature, but antifungal treatment is usually discontinued once the lesions have resolved and fungal cultures became negative [3, 7].

Treatment used in the present case was based on the guidelines published by the European Society of Clinical Microbiology and Infectious Diseases (ESCMID) and the European Confederation of Medical Mycology (ECMM) for the diagnosis and management of rare invasive yeast infections [2]. Treatment with voriconazole and terbinafine was administered for two months, but a rapid relapse occurred following discontinuation. Subsequently itraconazole and fluconazole were administered without clinical improvement; likely reflecting the limited efficacy of these antifungal agents against *Sporobolomyces roseus* [11].

A limitation of this case report was the absence of visible fungal elements in the histopathological sections, despite the use of PAS, and GMS special stains, indicating that these stains should not be solely relied upon to exclude the differential of fungal infection. Nonetheless, not all reported cases in human medicine are based on histopathological diagnosis. In some instances, the diagnosis relies solely on a positive fungal culture for *Sporobolomyces* spp [3, 7]. For example, in the case reported by Damji et al., histopathological examination using HE stains did not reveal any fungal elements, but *Sporobolomyces salmonicolor* was grown on fungal culture [3].

In this case, the definitive diagnosis relied exclusively on a positive pan-fungal PCR result. Ideally, fungal culture from biopsy material should have been performed to confirm the etiological role of *Sporobolomyces roseus*. Consequently, a definitive causal relationship between *Sporobolomyces roseus* and the dermatological lesions in this dog cannot be absolutely confirmed but is strongly supported by the initial clinical response to antifungal therapy.

In conclusion, this case report described for the first time the dermatological lesions associated with the identification of *Sporobolomyces roseus* by PCR on skin biopsy specimens in a dog.

The prognosis of this fungal infection in veterinary medicine appears guarded to poor, particularly when financial constraints limit the prolonged use of antifungal drugs.

## Data Availability

No datasets were generated or analysed during the current study.

## Abbreviations

PCR: Polymerase chain reaction
ITS: Internal transcribed spacer
HE: Hematoxylin and eosin
PAS: Periodic acid shiff
GMS: Grocott’s Methenamine Silver
ESCMID: European society of clinical microbiology and infectious diseases
ECMM: European confederation of medical mycology
